# What is considered cardiotoxicity of anthracyclines in animal studies

**DOI:** 10.3892/or.2020.7717

**Published:** 2020-08-06

**Authors:** Nikolaos Georgiadis, Konstantinos Tsarouhas, Ramin Rezaee, Haritini Nepka, George E.N. Kass, Jean-Lou C.M. Dorne, Dimitrios Stagos, Konstantinos Toutouzas, Demetrios A. Spandidos, Dimitrios Kouretas, Christina Tsitsimpikou

Oncol Rep 44: 798-818, 2020; DOI: 10.3892/or.2020.7688

Subsequently to the publication of this paper, the authors have realized that the name of the seventh listed author, Dimitrios Stagos, was spelt incorrectly (it appeared as ‘Stagkos’ in print). The corrected author list is shown above. The authors regret that the name of the seventh author on the paper was spelt incorrectly, and apologize to the readers for any inconvenience caused.

